# *RFX3* Pathogenic Variants as a Rare Cause of Infantile Epileptic Spasms Syndrome

**DOI:** 10.3390/ijms27167238

**Published:** 2026-08-13

**Authors:** Graziana Ceraolo, Giulia Spoto, Marina Trivisano, Concetta Federico, Mirella Vinci, Francesco Calì, Simone Treccarichi, Antonino Musumeci, Gabriella Di Rosa, Nicola Specchio, Antonio Gennaro Nicotera

**Affiliations:** 1 Unit of Child Neurology and Psychiatry, Department of Human Pathology of the Adult and Developmental Age “Gaetano Barresi”, University of Messina, 98125 Messina, Italy; graziana.c23@hotmail.it; 2 Unit of Child Neurology and Psychiatry, Department of Biomedical Sciences, Dental Sciences & Morpho—Functional Imaging, University of Messina, 98125 Messina, Italy; giulia.spoto27@gmail.com (G.S.); gdirosa@unime.it (G.D.R.); 3 IRCCS Centro Neurolesi Bonino-Pulejo, 98124 Messina, Italy; 4 Neurology, Epilepsy and Movement Disorders Unit, Bambino Gesu’ Children’s Hospital IRCCS, EpiCARE, 00168 Rome, Italy; marina.trivisano@opbg.net (M.T.); nicola.specchio@opbg.net (N.S.); 5 Department of Biological, Geological and Environmental Sciences, University of Catania, 95124 Catania, Italy; concetta.federico@unict.it; 6 Oasi Research Institute—IRCCS, 94018 Troina, Italy; mvinci@oasi.en.it (M.V.); cali@oasi.en.it (F.C.); amusumeci@oasi.en.it (A.M.); 7 Department of Medicine and Surgery, Kore University of Enna, 94100 Enna, Italy; 8 Unit of Child Neurology and Psychiatry, Maternal-Infantile Department, University of Messina, 98125 Messina, Italy; antonionicotera@ymail.com

**Keywords:** infantile epileptic spasms syndrome, *RFX3*, DEE, autism spectrum disorder, haploinsufficiency, seizure

## Abstract

Regulatory Factor X3 (RFX3—OMIM#601337) encodes a transcription factor that is highly expressed in the human brain, particularly during neurodevelopment. It has been previously associated with neurodevelopmental disorders, including autism spectrum disorder (ASD), intellectual developmental disorder, and attention-deficit/hyperactivity disorder. However, the neurological and epileptic features remain poorly characterized, and no phenotype has yet been formally annotated in OMIM. Here, we report the second known case of Infantile Epileptic Spasms Syndrome (IESS) associated with *RFX3* variants. The patient developed clusters of extensor spasms associated with eye deviation and achieved complete remission within two weeks following vigabatrin and ACTH therapy, remaining seizure-free thereafter. During follow-up, he presented with global developmental delay, ASD, and facial dysmorphisms. Genetic analysis by array comparative genomic hybridization identified a de novo heterozygous microdeletion of approximately 147 kb at 9p24.2, involving the initial exons of *RFX3* (NM_134428). This case expands the clinical spectrum associated with *RFX3* variants, supporting a potential role in IESS and early neurodevelopmental disruption. It highlights the relevance of including *RFX3* in the genetic evaluation of patients with IESS and co-occurring neurodevelopmental disorders.

## 1. Introduction

Regulatory Factor X3 (RFX3—OMIM#601337) is located in the region 9p24.2 and encodes a transcription factor belonging to the regulatory factor X (RFX) family, characterized by a highly conserved winged-helix DNA-binding domain that recognizes X-box motifs in cis-regulatory regions [1]. RFX3 functions as a transcriptional activator, binding DNA either as a monomer or as a heterodimer with other RFX family members [2]. *RFX3* is expressed in multiple tissues, with particularly high expression in the developing and adult human brain, where it plays a fundamental role in the regulation of ciliogenesis by controlling the expression of multiple genes involved in cilia assembly and function [3,4]. A mouse model showed that *RFX3* is initially expressed throughout the anterior neural tube during early neurodevelopment, and subsequently becomes restricted to specific cell populations, particularly at the midline of the corticoseptal boundary, supporting a key role in cortical patterning and maturation [3].

Pathogenic variants in *RFX3*—most commonly de novo loss-of-function variants—have been associated with a neurodevelopmental phenotype characterized by Autism Spectrum Disorder (ASD), Intellectual Developmental Disorder (IDD) or global developmental delay, attention-deficit/hyperactivity disorder (ADHD), and distinctive behavioural features including sensory hypersensitivity (especially auditory), emotional dysregulation, anxiety, and impulsivity, typically in the absence of specific abnormalities on neuroimaging [2].

However, while the neurodevelopmental phenotype has been relatively well described, the neurological and epileptic manifestations associated with *RFX3* variants remain poorly characterized. Seizures have been reported in 17% of affected individuals, suggesting a possible association between *RFX3* variants and neuronal hyperexcitability [2]. In 2023, Torio and colleagues reported the first case of infantile epileptic spasms syndrome (IESS) in a child with a de novo pathogenic variant in *RFX3*, in association with ASD and IDD [5].

IESS is a developmental and epileptic encephalopathy (DEE) of infancy occurring during a critical window of brain development [6]. IESS arises from diverse genetic, structural, metabolic, and acquired factors that converge on abnormal network maturation and neuronal hyperexcitability, representing a common downstream mechanism [7,8].

Here, we describe the second known case of IESS associated with a de novo 9p24.2 microdeletion involving the RFX3 gene, thereby contributing to the expanding clinical and phenotypic characterization of *RFX3*-related neurodevelopmental disorders.

## 2. Results

### 2.1. Case Report

**Figure 1:**
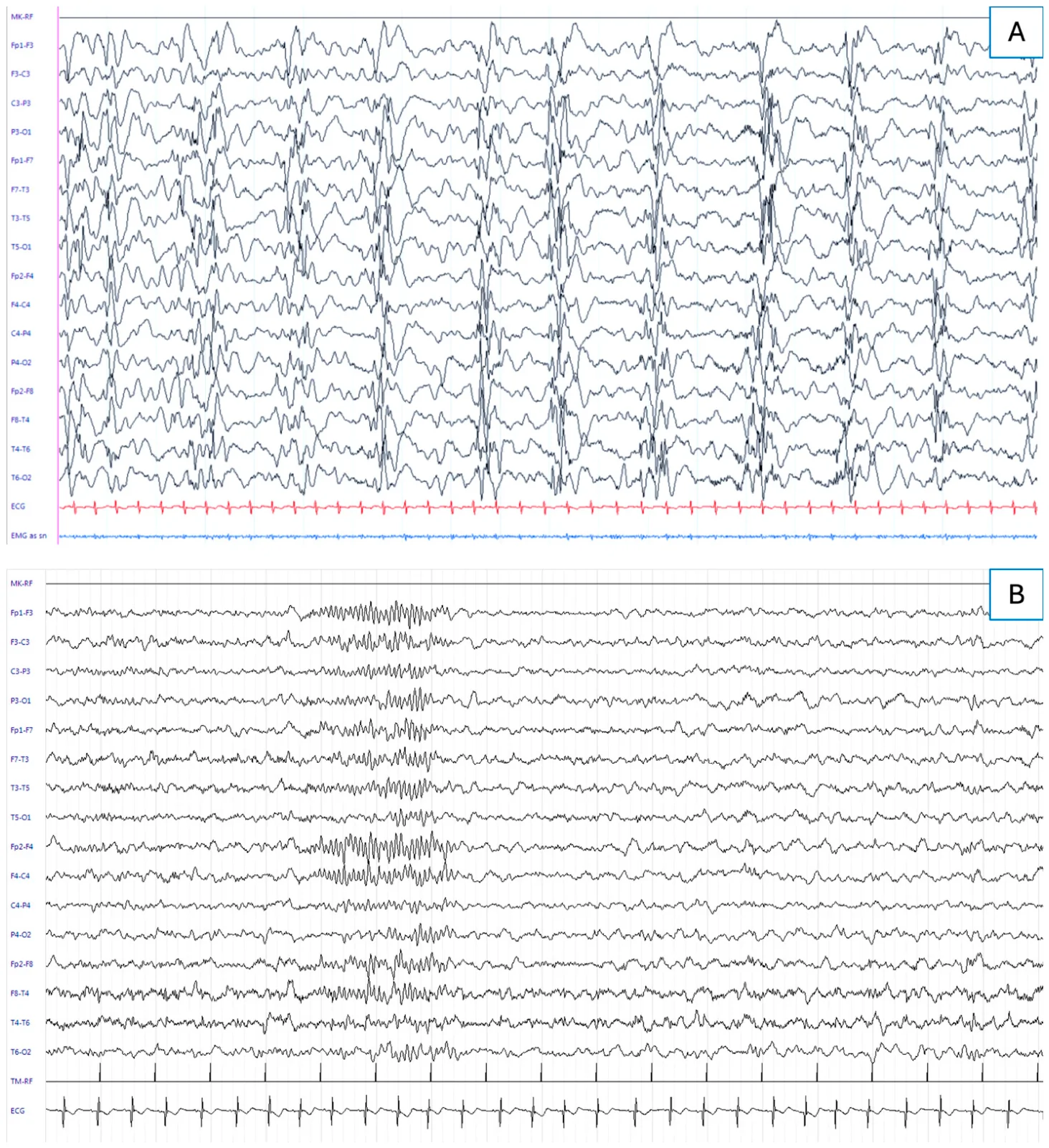
Pre-and post-treatment sleep EEG recordings: (**A**). Pre-treatment EEG showing a severe encephalopathic pattern, characterized by disorganized background activity and multifocal high-amplitude epileptiform discharges. (**B**). Sleep EEG after 6 months of ACTH therapy, demonstrating normal organization of background activity, absence of epileptiform abnormalities and preserved physiological sleep features.

We report a 3-year-old Caucasian boy, the first child of healthy, non-consanguineous parents. There was no family history of epilepsy and other neurological disorders, except for a maternal uncle with febrile seizures. He was born at full term by uncomplicated vaginal delivery after a pregnancy characterized by threatened miscarriage at 5 months, treated with progestins. At 7 months of age, the patient was referred to our department due to the onset of epileptic extensor spasms, occurring in clusters and occasionally lasting longer than 5 min, associated with upward eye deviation. The child showed concomitant behavioural changes, including a reduced frequency of smiling and failure to achieve independent sitting. On general examination, mild facial dysmorphic features were noted, including a long face, broad forehead, flat nasal bridge, hypertelorism and epicanthal folds. Neurological examination revealed mild axial hypotonia. Routine blood tests were unremarkable, with the exception of hypogammaglobulinemia; urine tests and all other instrumental investigations (among which echocardiogram and abdominal ultrasound) were within normal limits. Brain magnetic resonance imaging (MRI) revealed no structural abnormalities. Developmental assessment using the Griffiths Mental Development Scales—Revised (GMDS-R) revealed a General Quotient of 77, resulting in mild global developmental delay. Interictal electroencephalogram (EEG) showed high-voltage slow waves and multifocal spikes, consistent with hypsarrhythmia (Figure 1). Ictal EEG showed generalized, irregular high-voltage slow waves. A diagnosis of IESS was established and treatment with vigabatrin was initiated (up to 120 mg/kg/day), resulting in a partial reduction in spasms. After approximately one month, due to increased frequency and intensity of the spasms, adrenocorticotropic hormone (ACTH) depot therapy was administered. Epileptic spasms and EEG abnormalities resolved within two weeks of ACTH initiation. At the most recent follow-up evaluation, at 3 years of age, the patient remained seizure-free, with no recurrence of epileptic spasms. Neurological examination revealed only mild diffuse hypotonia, without other abnormalities. Despite the favourable seizure outcome, developmental difficulties persisted, especially in communication and language domains. Expressive language was limited to simple sentences and was characterized by immediate and delayed echolalia. Clinical features suggestive of ASD—such as poor eye contact, stereotyped behaviours, and atypical sensory responsiveness—emerged over time and ultimately led to a diagnosis of ASD at the most recent follow-up. The diagnosis was further supported by the administration of the Autism Diagnostic Observation Schedule, Second Edition (ADOS-2), Module 1, which yielded a total score of 16, consistent with autism. Developmental assessment using the GMDS—Extended Revised yielded a General Quotient of 90.5, within the age-appropriate range.

### 2.2. Genetic Analysis

**Figure 2:**
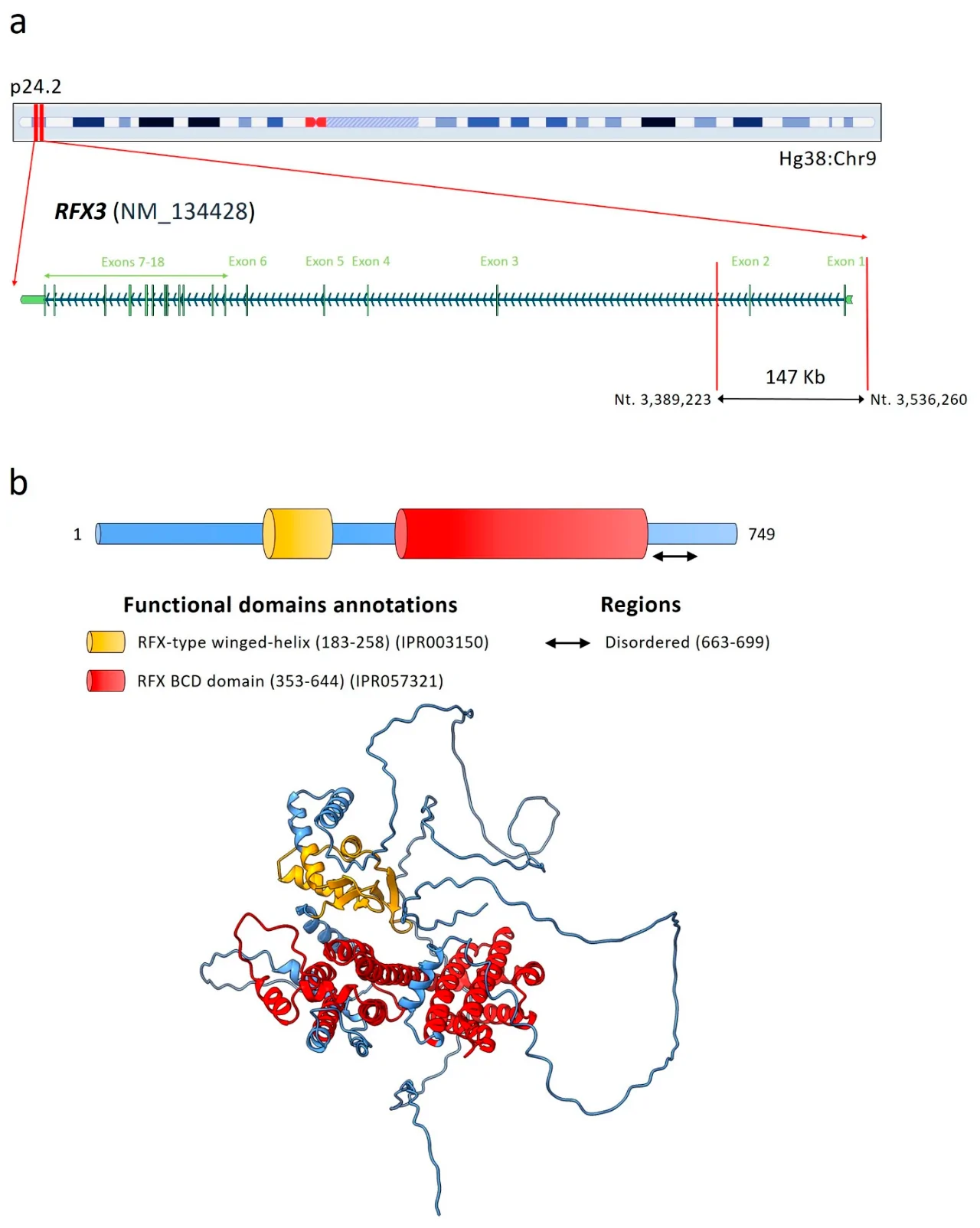
Structural overview of the RFX3 gene and protein, and localization of the identified microdeletion. (**a**) Genomic organization of the *RFX3* (NM_134428) located on chromosome 9p24.2. The schematic representation shows the exon–intron structure, with exons labeled from 1 to 18. The position of the translation start site (AUG) is indicated. The red bar highlights the heterozygous deletion identified by array-CGH, spanning approximately 147 kb (chr9: 3389223-3536260), encompassing part of the RFX3 locus. (**b**) Schematic representation of the RFX3 protein, including annotated functional domains and structural features. The RFX-type winged-helix DNA-binding domain (IPR003150) and the RFX BCD domain (IPR057321) are indicated. A predicted intrinsically disordered region (residues 663–699) is also shown.

An epilepsy-targeted NGS panel did not identify pathogenic variants. Conversely, CGH-array analysis revealed a microdeletion involving 9p24.2, approximately 147 kb in size, encompassing exons 1 and 2 of *RFX3* (NM_134428) gene (Figure 2). Analysis of both parents demonstrated that neither carried the microdeletion, confirming the variant as de novo. According to ACMG criteria, the identified CNV is classified as likely pathogenic primarily driven by overlap with a haploinsufficient genomic region (see Table 1).

### 2.3. Exploratory Analysis of RFX3 Expression in the GSE186334 Dataset

To investigate whether RFX3 expression is altered in human epileptic brain tissue, we analyzed the publicly available RNA-sequencing dataset GSE186334, including hippocampal and cortical samples from patients with mesial temporal lobe epilepsy (mTLE) and post-mortem controls. Separate analyses were performed for cytoplasmic and nuclear RNA fractions using DESeq2. *RFX3* expression was readily detectable across all tissues and fractions analyzed (Figure S1). However, no statistically significant differential expression was observed between mTLE non-HS samples and post-mortem controls in either hippocampal cytoplasmic or nuclear fractions. Likewise, no significant differences were detected in cortical samples from patients with mTLE non-HS or mTLE + HS compared with post-mortem controls (Table S1). Overall, these findings indicate that *RFX3* transcript abundance is preserved in adult epileptic brain tissue despite the presence of chronic epilepsy-associated pathology.

## 3. Discussion

To date, pathogenic variants in *RFX3* have been associated with a neurodevelopmental disorder characterized by ASD, developmental impairment, ADHD, and behavioural dysregulation (see Table 2). In 2021, Harris and colleagues reported a cohort of 18 individuals carrying pathogenic *RFX3* variants. The most prevalent clinical diagnoses included ASD (72%), IDD or global developmental delay (78%), and ADHD (56%). Additional features included nonspecific dysmorphic traits (61%), such as broad nasal bridge, high-arched palate, macro- or microcephaly, and hand and foot abnormalities (e.g., tapered fingers, widely spaced toes), hypotonia (approximately 50%), sleep difficulties (44%), and seizures (17%). Beyond formal diagnoses of ASD and ADHD, a distinctive behavioural profile has been described, including sensory hypersensitivity and impulsivity, often associated with hyperexcitability, anxiety, and emotional dysregulation [2]. Although seizures were reported in 17% of individuals, detailed information about seizures characteristics, EEG findings, and treatment were not provided, precluding characterization of the epilepsy phenotype [2]. In 2023, Torio and colleagues described an additional case of an *RFX3* pathogenic variant presenting with IESS responsive to ACTH, persistent neurodevelopmental delay, and facial dysmorphisms [5].

In line with this, our patient presented with developmental delay, ASD, IESS, and facial dysmorphisms largely overlapping with those reported by Torio and colleagues [5]. Both cases of IESS showed a favourable response to ACTH therapy, achieving good seizure control and remaining consistently seizure-free during follow-up. The efficacy of steroid therapy in epileptic spasms is well recognized. Current ILAE recommendations indicate ACTH as the preferred treatment for short-term spasm control [9,10]. With regard to EEG findings, both cases exhibited hypsarrhythmia at presentation. In our patient, EEG abnormalities resolved following clinical improvement of seizures. Notably, despite the overlapping clinical phenotype, the variant reported by Torio and colleagues is a missense variant, whereas our case carries a de novo microdeletion involving the first coding exons (exons 1–2) of *RFX3*, likely leading to a loss-of-function effect due to the predicted absence of a functional protein.

According to publicly available datasets, including DECIPHER, multiple overlapping CNVs involving this genomic region have been reported, predominantly deletions, with a substantial proportion occurring de novo. This recurrence pattern supports the clinical relevance of dosage alterations affecting this locus. Among the genes included in the deleted interval, only *RFX3* and its divergent transcript (RFX3-DT) are encompassed. Notably, RFX3-DT represents a non-coding transcript with no established disease association. Therefore, *RFX3* emerges as the most plausible disease-associated candidate, supported by multiple, converging lines of evidence indicating strong dosage sensitivity. *RFX3* displays a pHaplo score of 1.00, exceeding the established threshold (≥0.86) for deletion effect sizes comparable to those observed for genes highly constrained against protein-truncating variants. Consistently, additional constraint metrics (pLI = 1.00; LOEUF = 0.21) further indicate marked intolerance to loss-of-function variation. Moreover, ClinGen and OMIM annotations support an autosomal dominant inheritance pattern, reinforcing the concept of dosage sensitivity for this gene. Importantly, the deletion involves the 5′ portion of *RFX3*, including its initial exons, a region critical for gene expression and transcript integrity. Such a genomic configuration is highly consistent with a loss-of-function effect, either through disruption of transcription initiation or through generation of a non-functional transcript. Taken together, these observations strongly support haploinsufficiency of *RFX3* as the most plausible pathogenic mechanism underlying the observed genomic alteration.

**Figure 3:**
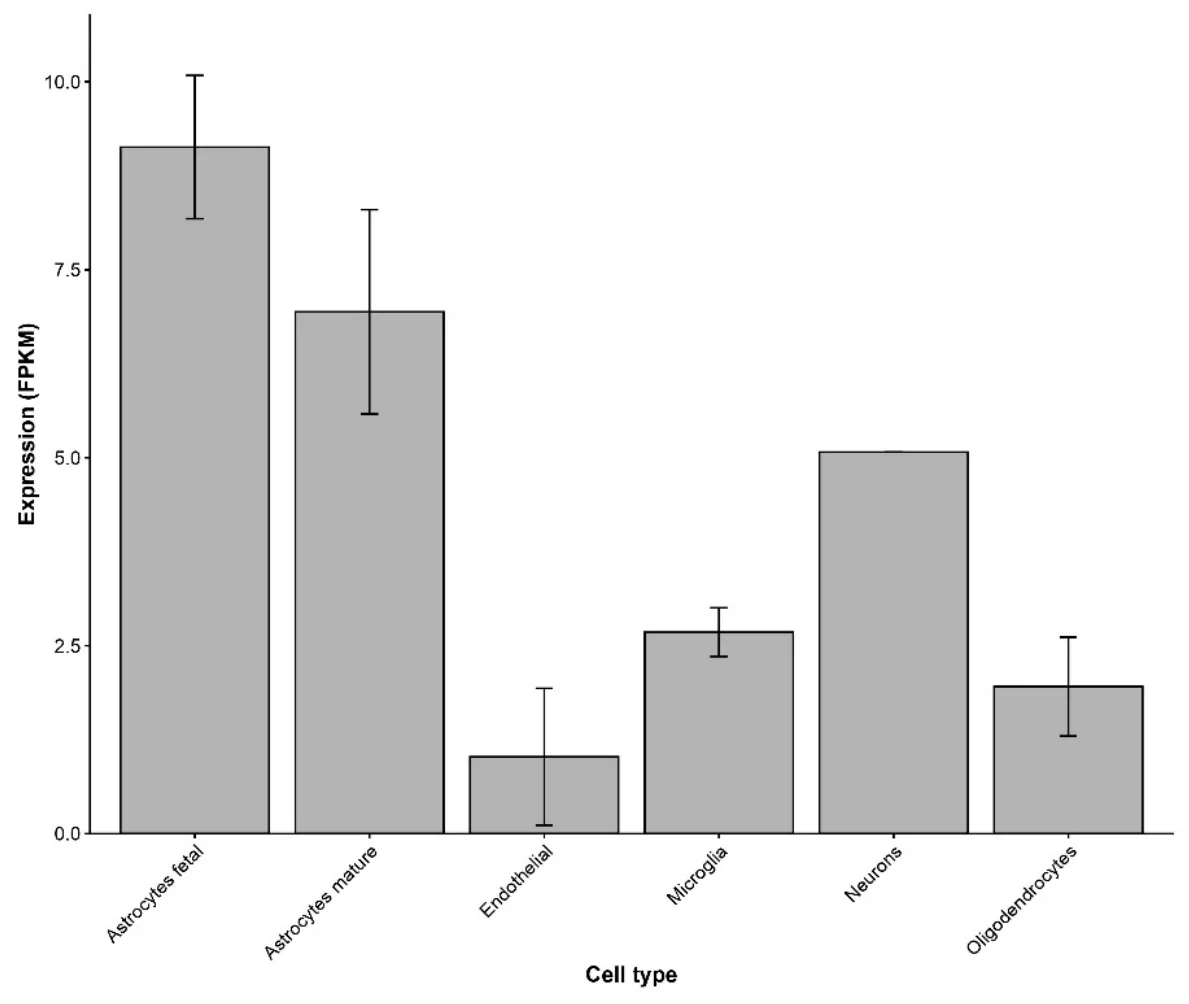
Cell type-specific expression of RFX3 in the human brain. RFX3 expression (FPKM) across major human brain cell types was retrieved from the BrainRNAseq database. The dataset comprises transcriptomic profiles from non-diseased human brain samples, including foetal astrocytes (n = 6), mature astrocytes (n = 12), endothelial cells (n = 2), microglia (n = 3), neurons (n = 1), and oligodendrocytes (n = 5). n indicates the number of samples available in the BrainRNAseq database for each cell type. Error bars represent standard deviation (SD) where applicable. No error bar is shown for neurons because the BrainRNAseq dataset contains a single neuronal sample. The BrainRNAseq dataset has been previously described [13].

*RFX3* is highly expressed in upper-layer cortical projection neurons, which are crucial for long-range cortico-cortical communication underlying higher cognitive and social functions [11]. Consistently, epigenomic studies have reported enrichment of RFX binding motifs within ASD-upregulated acetylation peaks in both the temporal and prefrontal cortices, supporting a role for *RFX3* in the regulation of transcriptional programs relevant to cortical development and neuronal excitability, further suggesting its involvement as a risk gene for ASD [2,12]. According to the BrainRNAseq database, *RFX3* is broadly expressed across multiple neural cell types, with prominent expression in neurons and astrocytes (Figure 3).

**Figure 4:**
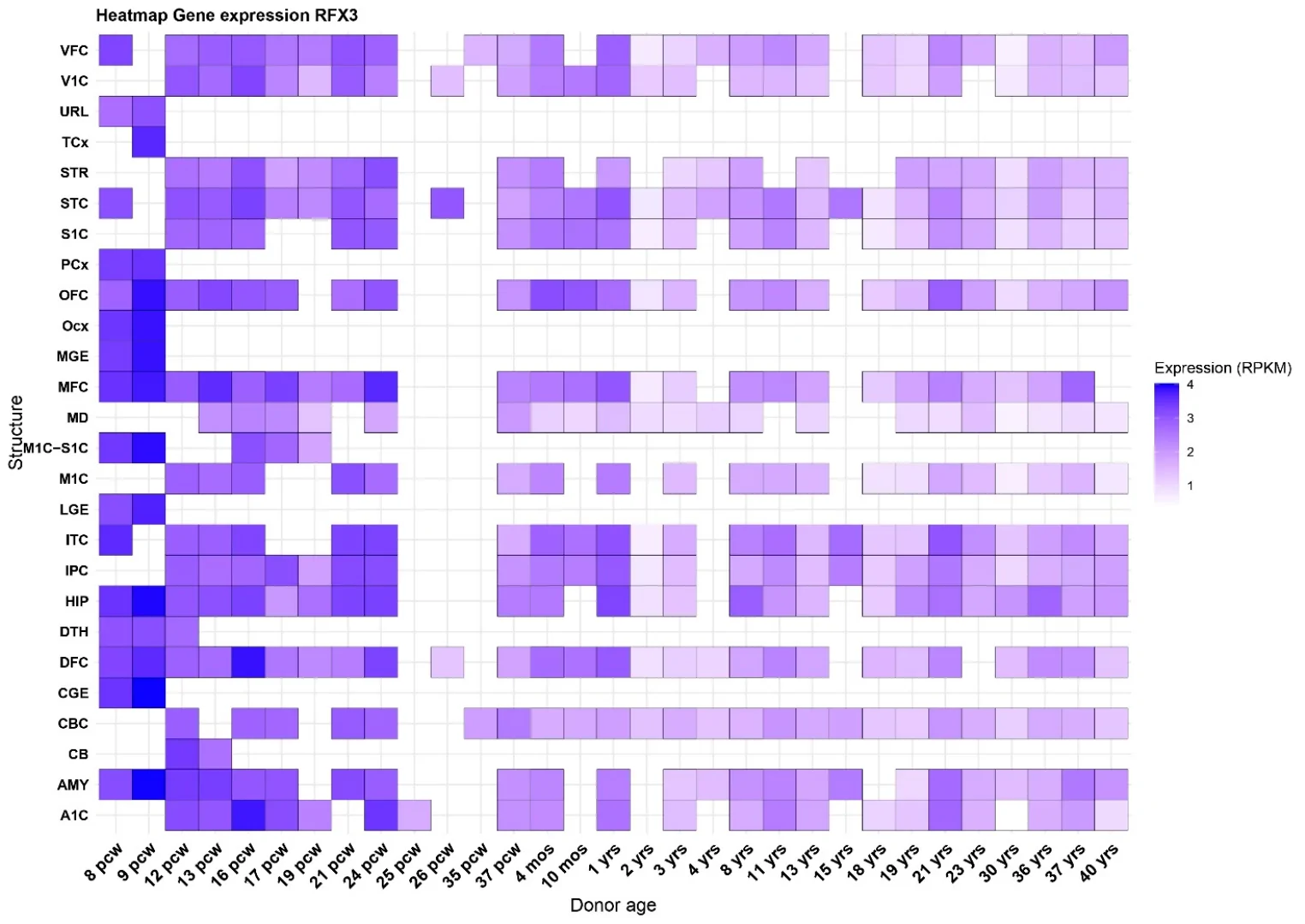
Developmental profile of *RFX3* in human brain across the lifespan. Data were retrieved from BrainSpan database. The abbreviation used were: primary auditory cortex (core) (A1C); amygdaloid complex (AMY); cerebellum (CB); cerebellar cortex (CBC); caudal ganglionic eminence (CGE); dorsolateral pre-frontal cortex (DFC); dorsal thalamus (DTH); hippocampus (hippocampal formation) (HIP); posteroventral (inferior) parietal cortex (IPC); inferolateral temporal cortex (area TEv, area 20) (ITC); lateral ganglionic eminence (LGE); primary motor cortex (area M1, area 4) (M1C); primary motor-sensory cortex (samples) (M1C-S1C); mediodorsal nucleus of thalamus (MD); anterior (rostral) cingulate (medial prefrontal) cortex (MFC); medial ganglionic eminence (MGE); occipital neocortex (Ocx); orbital frontal cortex (OFC); parietal neocortex (PCx); primary somatosensory cortex (area S1, areas 3,1,2) (S1C); posterior (caudal) superior temporal cortex (area 22c) (STC); striatum (STR); temporal neocortex (TCx); upper (rostral) rhombic lip (URL); primary visual cortex (striate cortex, area V1/17) (V1C); ventrolateral prefrontal cortex (VFC); post-conceptional week (pcw); months (mos); years (yrs).

Notably, expression levels are higher in foetal astrocytes compared to mature cells, suggesting a role in early glial development and brain maturation. Consistent with these findings, data from the BrainSpan database, which captures spatiotemporal gene expression patterns, show that *RFX3* is widely expressed across several brain regions, with peak expression during prenatal stages (Figure 4).

This developmental profile indicates a critical role for *RFX3* during early brain development, a period characterized by tightly regulated processes such as neuronal differentiation and circuit formation. The preferential expression of *RFX3* in the developing brain, together with its marked intolerance to loss-of-function variation and high haploinsufficiency score, provides strong functional support for its involvement in neurodevelopmental phenotypes. In this context, haploinsufficiency of *RFX3*, as observed in our patient, is likely to disrupt finely regulated developmental pathways, thereby contributing to the clinical presentation. These expression patterns are consistent with previously reported phenotypes associated with *RFX3* alterations and further reinforce its role as the primary disease-associated gene within the deleted interval. Evidence from Rfx3-deficient mouse models further highlights its critical role in early brain development, showing impaired neuronal migration and abnormal maturation of cortical and interhemispheric connectivity, including defects of the corpus callosum and thalamocortical tracts [14]. In addition, impaired differentiation and function of ciliated ependymal cells support the involvement of *RFX3* in ciliopathy-related mechanisms that may result in hydrocephalus [15]. Consistently, corpus callosum abnormalities have been linked to disrupted midline patterning and guidepost neuron organization, driven by altered cilia-dependent *GLI3* processing and focal *FGF8* dysregulation secondary to Rfx3 deficiency [3].

Recent studies using human stem cell–derived models demonstrate that RFX3, together with its likely target transcription factor RFX4, is expressed in developing cortical interneurons and regulates interneuron and excitatory neuron development, restraining premature interneuron differentiation while supporting cortical maturation [16]. Consequently, RFX3 deficiency leads to early upregulation of genes involved in neurogenesis and neuronal differentiation, potentially resulting in aberrant maturation of inhibitory interneurons and disruption of the excitation–inhibition balance within cortical circuits [15].

This network imbalance provides a biologically plausible mechanism linking RFX3 deficiency to increased neuronal excitability and susceptibility to early-onset epileptic phenotypes, including IESS. In this context, although most previously reported *RFX3*-related cases involve truncating or splice-site variants, our finding further supports haploinsufficiency as the primary disease mechanism, potentially contributing to seizure susceptibility through impaired cortical network integration and increased neuronal excitability.

To provide additional biological context for the identified RFX3 deletion, we analysed an independent publicly available RNA-sequencing dataset derived from patients with mesial temporal lobe epilepsy. Although RFX3 was consistently expressed across hippocampal and cortical tissues, no significant differences in transcript abundance were observed between epileptic and control samples. These findings should be interpreted with caution. The analysed dataset represents transcriptomic profiles obtained from surgically resected adult epileptic tissue, whereas the genetic alteration identified in our patient is a constitutional de novo deletion predicted to result in *RFX3* haploinsufficiency during neurodevelopment. Consequently, the absence of differential expression in adult epileptic tissue does not exclude a pathogenic role for germline RFX3 deficiency during early brain development. Moreover, mesial temporal lobe epilepsy and infantile epileptic spasms syndrome represent distinct clinical entities with different developmental timing and underlying pathophysiological mechanisms. Therefore, this exploratory transcriptomic analysis should not be considered a functional validation of the identified deletion. Rather, it demonstrates that RFX3 is expressed in epilepsy-relevant human brain regions and provides independent transcriptomic evidence from disease-associated tissue, providing additional transcriptomic context from disease-relevant human brain tissue.

Although the identified CNV is a plausible explanation for the observed phenotype, the absence of comprehensive NGS analyses, such as whole-exome sequencing or whole-genome sequencing, represents a limitation of this study. These approaches could have identified additional single-nucleotide variants that may contribute to the phenotype, either as primary drivers or as modifying factors [17,18,19]. The patient underwent targeted NGS analysis using a panel of 26 epilepsy-associated genes; however, no pathogenic or likely pathogenic variants were identified. Nevertheless, given the limited gene set analysed, this approach does not allow a comprehensive assessment of the genetic architecture of epilepsy, and the presence of additional contributing variants cannot be excluded. Accordingly, the proposed genotype–phenotype correlation should be interpreted with appropriate caution. Despite these limitations, and based on the analyses performed, the de novo microdeletion encompassing *RFX3* remains the most compelling candidate to explain the patient’s phenotype. An additional limitation of the present study is the absence of functional validation using patient-derived biological material. Although we complemented our genetic findings by interrogating an independent public RNA-sequencing dataset from human epileptic brain tissue, these analyses remain exploratory and cannot directly demonstrate the functional consequences of the identified RFX3 deletion. Future transcriptomic and cellular studies using patient-derived samples will be required to establish the molecular mechanisms underlying RFX3 haploinsufficiency.

In conclusion, we report the second known case of IESS associated with *RFX3* variants, providing further clinical evidence supporting a role for *RFX3* haploinsufficiency in early-onset DEEs within a broader neurodevelopmental spectrum. The favourable response to ACTH and sustained seizure freedom observed in both reported cases may suggest a favourable epileptic outcome, particularly considering that IESS may evolve into other DEEs, although further evidence is needed. Early recognition of autistic features is especially important, as timely behavioural and developmental interventions may significantly improve adaptive, communicative, and social outcomes, reinforcing the value of prompt genetic diagnosis and multidisciplinary management in individuals with *RFX3*-related disorders [20,21]. Overall, these findings expand the phenotypic spectrum of *RFX3*-related disorders and support the inclusion of *RFX3* in the genetic evaluation of patients with IESS and co-occurring neurodevelopmental disorders, while highlighting the need for further studies to clarify its role in epileptogenesis.

## 4. Materials and Methods

### 4.1. CGH-Array Analysis and Target Gene Panel

Genomic DNA was extracted from peripheral blood of the proband and both parents. CGH-array analysis was performed using an Illumina CytoSNP-850K array in a trio-based approach, with an average resolution of ~100 kb. Data were analyzed with BlueFuse Multi Software (v4.5; GRCh38), and Copy Number Variants (CNVs) were called based on ≥10 consecutive probes. Detected variants were confirmed by Agilent 4 × 180K CGH array. Targeted next-generation sequencing (NGS) was performed in a trio-based approach using the ClinEx Pro kit (4bases) on the Illumina NovaSeq 6000 platform. The analysis was focused on coding regions and exon–intron boundaries (±5 bp) of a panel of epilepsy-associated genes, including KCNQ2 (NM_172107), KCNQ3 (NM_004519), SCN2A (NM_021007), SCN8A (NM_014191), PCDH19 (NM_001184880), SCN3A (NM_006922), SCN9A (NM_002977), PRRT2 (NM_145239), SCN1A (NM_001165963), DEPDC5 (NM_001242896), KCNMA1 (NM_001161352), CHRNA2 (NM_000742), CHRNA4 (NM_000744), CHRNB2 (NM_000748), SCN1B (NM_001037), GRIN2A (NM_000833), GABRG2 (NM_198903), SLC2A1 (NM_006516), GABRD (NM_000815), SLC6A1 (NM_003042), STXBP1 (NM_003165), HCN1 (NM_021072), KCNT1 (NM_020822), NPRL2 (NM_006545), NPRL3 (NM_001077350), and SLC12A5 (NM_020708). The assay achieved an analytical sensitivity and specificity >99%, with a mean coverage of 245×. Only regions with a minimum read depth of 30× were considered for variant interpretation.

### 4.2. Data Analysis

Franklin by Genoox (https://franklin.genoox.com/) (accessed on 25 March 2026) was used to classify the identified CNVs according to the American College of Medical Genetics and Genomics (ACMG) criteria [22]. The analysis was performed using the following query: “arr[GRCh38] 9p24.2(3389223_3536260)x1”. DECIPHER database (https://www.deciphergenomics.org/) (accessed on 25 March 2026) was used to compare the genomic region interested by the CNVs found in this study with those previously reported involving the same region. The query for the DECIPHER research was: “9:3389223-3536260”. BrainRNAseq database (https://brainrnaseq.org/) (accessed on 25 March 2026) was used to study the expression profile of RFX3 across different human brain cells. BrainSpan database (https://www.brainspan.org/) (accessed on 25 March 2026) was used to study the developmental brain expression of *RFX3*. THE Uniprot (https://www.uniprot.org/) database (accessed on 25 March 2026) was used to study the functional annotations related to the RFX3 protein, with the entry code P48380.

### 4.3. Transcriptomic Analysis of Epilepsy-Relevant Human Brain Tissue

To further investigate *RFX3* expression in disease-relevant human brain tissue, we analyzed the publicly available RNA-sequencing dataset GSE186334 obtained from the Gene Expression Omnibus (GEO) (https://ncbi.nlm.nih.gov/geo/) (accessed on 2 August 2026). The dataset comprises hippocampal and cortical samples from patients with mesial temporal lobe epilepsy (mTLE), including individuals with and without hippocampal sclerosis (HS), as well as post-mortem controls. Cytoplasmic and nuclear RNA fractions were analyzed separately. Raw gene count matrices and sample metadata were downloaded from GEO. Differential expression analysis was performed using the **DESeq2** package in R studio (version 4.5.2). Analyses were conducted separately for each tissue (hippocampus and cortex) and RNA fraction (cytoplasm and nucleus). Cortical samples were compared between (i) mTLE without hippocampal sclerosis (mTLE non-HS) and post-mortem controls and (ii) mTLE with hippocampal sclerosis (mTLE + HS) and post-mortem controls. For hippocampal tissue, only the comparison between mTLE non-HS and post-mortem controls was performed, as hippocampal samples from patients with HS were not available. Differential expression was assessed using the standard DESeq2 workflow with Wald statistics and Benjamini–Hochberg correction for multiple testing. DESeq2-normalized counts were used for graphical visualization.

## Figures and Tables

**Table 1:** ACMG criteria used for classifying the chr9:3389223-3536260 CNV identified in this study.

Category	Criterion	Description
Genomic content	1A	CNV contains protein-coding or functionally relevant elements
Dosage sensitivity	2A–2E	Partial overlap with an established haploinsufficient (HI) gene/region; CNV does not fully include the critical region
Gene number	3A	Number of protein-coding genes within the CNV region (0–24 genes)
Inheritance	5A	De novo occurrence

**Table 2:** Genotypic and phenotypic features of individuals with *RFX3* variants.

References	Age, Gender	*RFX3* Variants	Inheritance	DD/IDD	ASD	ADHD	Seizures	Neurobehavioral Features	Dysmorphic Features	HC	Brain Imaging
Harris et al., 2021 [2]	20y, M	c.584_586del; p.(Glu195del)	de novo	Yes	Yes	Yes	No	Speech delay, hypotonia, anxiety, aggression, sensory hypersensitivity, stereotypies, sleep difficulties	Yes	Normal	NA
Harris et al., 2021 [2]	26y, M	c.722T > G; p.(Leu241Trp)	de novo	Yes	Yes	Yes	No	Speech delay, hypotonia, sensory-seeking behaviour, pica, aggression, SIB, sleep difficulties	Yes	Normal	Normal/ unspecific
Harris et al., 2021 [2]	10y, M	9p24.2 deletion	de novo	No	Yes	Yes	Yes	Sensory hypersensitivity, sleep difficulties	Yes	Macrocephaly	NA
Harris et al., 2021 [2]	7y, M	c.1968 + 8270_*27326del; p.Phe647_Val749del	de novo	Yes	Yes	Yes	No	Speech delay, anxiety	Yes	Mild macrocephaly	Normal/ unspecific
Harris et al., 2021 [2]	10y, F	c.1831G > T; p.(Asp611Tyr)	de novo	No	Yes	Yes	No	Oppositional behaviour, aggression	Yes	Mild macrocephaly	NA
Harris et al., 2021 [2]	12y, M	c.1704dup; p.(Trp569Valfs*6)	de novo	No	Yes	Yes	No	Hypotonia, anxiety, aggression, emotional dysregulation, sleep difficulties	No	Macrocephaly	PVL
Harris et al., 2021 [2]	6y, M	c.1968 + 1G > A; splice-site	de novo	Yes	No	NA	No	Speech delay, hypotonia, sensory-seeking behaviour, aggression, emotional dysregulation, sleep difficulties	No	Normal	Partially empty sella
Harris et al., 2021 [2]	33y, F	c.1486_1487del; p.(Leu496Alafs*7)	Inherited*	Yes	Yes	NA	NA	Speech delay	Yes	NA	NA
Harris et al., 2021 [2]	7y, M	c.1486_1487del; p.(Leu496Alafs*7)	Inherited*	Yes	Yes	Yes	No	Speech delay, aggression	No	NA	NA
Harris et al., 2021 [2]	6y, F	c.1486_1487del; p.(Leu496Alafs*7)	Inherited*	NA	NA	NA	NA	Speech delay	NA	NA	NA
Harris et al., 2021 [2]	5y, M	c.1486_1487del; p.(Leu496Alafs*7)	Inherited*	NA	NA	NA	NA	Speech delay	NA	NA	NA
Harris et al., 2021 [2]	12y, M	c.549 + 5G > A; slice-site	de novo	Yes	Yes	No	No	Speech delay, hypotonia, sensory hypersensitivity, sleep difficulties	Yes	Mild macrocephaly	Normal/ unspecific
Harris et al., 2021 [2]	4y, M	c.1327C > A; p.(Leu443Ile)	de novo	NA	NA	NA	Yes	Hypotonia, sleep difficulties	Yes	Microcephaly	Thin CC
Harris et al., 2021 [2]	31y, M	c.674T > C; p.(Phe225Ser)	de novo	NA	Yes	Yes	NA	Sleep difficulties	NA	NA	NA
Harris et al., 2021 [2]	16y, F	c.1148T > C; p.(Phe383Ser)	de novo	Yes	Yes	Yes	No	Speech delay, emotional dysregulation	Yes	Macrocephaly	Normal/ unspecific
Harris et al., 2021 [2]	31y, M	c.587G > A; p.(Gly196Glu)	de novo	Yes	Yes	No	Yes	Aggression, sleep difficulties	No	NA	Uncal asymmetry
Harris et al., 2021 [2]	9y, M	c.1523C > A; p.(Ala508Glu)	de novo	Yes	No	No	No	Speech delay	Yes	NA	NA
Harris et al., 2021 [2]	8y, F	c.1813A > G; p.(Ser605Gly)	de novo	Yes	Yes	Yes	No	Speech delay, aggression, emotional dysregulation, sleep difficulties	Yes	Mild macrocephaly	NA
Torio et al., 2023 [5]	4y, M	c.1141C > G; p.(Gln381Glu)	de novo	Yes	Yes	No	Yes, infantile spasms	Hypotonia, stereotypies	Yes	Normal	Normal/ unspecific
Our case	3y, M	9p24.2 deletion	de novo	Yes	Yes	Yes	Yes, infantile spasms	Speech delay, hypotonia, sensory hypersensitivity, stereotypies	Yes	Normal	Normal/ unspecific

Legend = *: affected siblings; ADHD: Attention-Deficit/Hyperactivity Disorder; ASD: Autism Spectrum Disorder; CC: corpus callosum; DD: Developmental Delay; F: female; HC: Head Circumference; IDD: Intellectual Developmental Disorder; M: male; NA: not available; PVL: Periventricular Leukomalacia; SIB: self-injury behaviour; y: years.

## Data Availability

All data relevant to this case report are included in the manuscript.

## Supplementary Materials

The following supporting information can be downloaded at: https://www.mdpi.com/article/10.3390/ijms27167238/s1.

## Abbreviations

The following abbreviations are used in this manuscript:

ACMG	American College of Medical Genetics and Genomics
ACTH	Adrenocorticotropic hormone
ADHD	Attention-Deficit/Hyperactivity Disorder
ASD	Autism Spectrum Disorder
CGH-array	Chromosomal Microarray
CNV	Copy Number Variant
DEE	Developmental and Epileptic Encephalopathy
EEG	Electroencephalogram
IDD	Intellectual Developmental Disorder
IESS	Infantile Epileptic Spasms Syndrome
MRI	Magnetic Resonance Imaging
NGS	Next-Generation Sequencing
RFX3	Regulatory Factor X3
RFX3-DT	RFX3 divergent transcript

## References

[1] Sugiaman-Trapman D., Vitezic M., Jouhilahti E.M., Mathelier A., Lauter G., Misra S., Daub C.O., Kere J., Swoboda P. (2018). Characterization of the human RFX transcription factor family by regulatory and target gene analysis. BMC Genom..

[2] Harris H.K., Nakayama T., Lai J., Zhao B., Argyrou N., Gubbels C.S., Soucy A., Genetti C.A., Suslovitch V., Rodan L.H. (2021). Disruption of RFX family transcription factors causes autism, attention-deficit/hyperactivity disorder, intellectual disability, and dysregulated behavior. Genet. Med. Off. J. Am. Coll. Med. Genet..

[3] Benadiba C., Magnani D., Niquille M., Morlé L., Valloton D., Nawabi H., Ait-Lounis A., Otsmane B., Reith W., Theil T. (2012). The ciliogenic transcription factor RFX3 regulates early midline distribution of guidepost neurons required for corpus callosum development. PLoS Genet..

[4] Bonnafe E., Touka M., AitLounis A., Baas D., Barras E., Ucla C., Moreau A., Flamant F., Dubruille R., Couble P. (2004). The transcription factor RFX3 directs nodal cilium development and left-right asymmetry specification. Mol. Cell. Biol..

[5] Torio M., Maeda K., Akamine S., Kawakami S., Matsubara Y., Miya F., Kato M., Kira R. (2023). A case of infantile epileptic spasms syndrome and autism spectrum disorder with an RFX3 mutation. Seizure.

[6] Zuberi S.M., Wirrell E., Yozawitz E., Wilmshurst J.M., Specchio N., Riney K., Pressler R., Auvin S., Samia P., Hirsch E. (2022). ILAE classification and definition of epilepsy syndromes with onset in neonates and infants: Position statement by the ILAE Task Force on Nosology and Definitions. Epilepsia.

[7] Innes E.A., Han V.X., Patel S., Farrar M.A., Gill D., Mohammad S.S., Dale R.C. (2025). Aetiopathogenesis of infantile epileptic spasms syndrome and mechanisms of action of adrenocorticotrophin hormone/corticosteroids in children: A scoping review. Dev. Med. Child Neurol..

[8] Snyder H.E., Jain P., RamachandranNair R., Jones K.C., Whitney R. (2024). Genetic Advancements in Infantile Epileptic Spasms Syndrome and Opportunities for Precision Medicine. Genes.

[9] Wilmshurst J.M., Gaillard W.D., Vinayan K.P., Tsuchida T.N., Plouin P., Van Bogaert P., Carrizosa J., Elia M., Craiu D., Jovic N.J. (2015). Summary of recommendations for the management of infantile seizures: Task Force Report for the ILAE Commission of Pediatrics. Epilepsia.

[10] Di Rosa G., Dicanio D., Nicotera A.G., Mondello P., Cannavò L., Gitto E. (2020). Efficacy of Intravenous Hydrocortisone Treatment in Refractory Neonatal Seizures: A Report on Three Cases. Brain Sci..

[11] Sorensen S.A., Bernard A., Menon V., Royall J.J., Glattfelder K.J., Desta T., Hirokawa K., Mortrud M., Miller J.A., Zeng H. (2015). Correlated gene expression and target specificity demonstrate excitatory projection neuron diversity. Cereb. Cortex.

[12] Sun W., Poschmann J., Cruz-Herrera Del Rosario R., Parikshak N.N., Hajan H.S., Kumar V., Ramasamy R., Belgard T.G., Elanggovan B., Wong C.C.Y. (2016). Histone Acetylome-wide Association Study of Autism Spectrum Disorder. Cell.

[13] Zhang Y., Sloan S.A., Clarke L.E., Caneda C., Plaza C.A., Blumenthal P.D., Vogel H., Steinberg G.K., Edwards M.S.B., Li G. (2016). Purification and characterization of progenitor and mature human astrocytes reveals transcriptional and functional differences with mouse. Neuron.

[14] Magnani D., Morlé L., Hasenpusch-Theil K., Paschaki M., Jacoby M., Schurmans S., Durand B., Theil T. (2015). The ciliogenic transcription factor Rfx3 is required for the formation of the thalamocortical tract by regulating the patterning of prethalamus and ventral telencephalon. Hum. Mol. Genet..

[15] Baas D., Meiniel A., Benadiba C., Bonnafe E., Meiniel O., Reith W., Durand B. (2006). A deficiency in RFX3 causes hydrocephalus associated with abnormal differentiation of ependymal cells. Eur. J. Neurosci..

[16] Chapman G., Determan J., Jetter H., Kaushik K., Prakasam R., Kroll K.L. (2024). Defining cis-regulatory elements and transcription factors that control human cortical interneuron development. iScience.

[17] Lindy A.S., Stosser M.B., Butler E., Downtain-Pickersgill C., Shanmugham A., Retterer K., Brandt T., Richard G., McKnight D.A. (2018). Diagnostic outcomes for genetic testing of 70 genes in 8565 patients with epilepsy and neurodevelopmental disorders. Epilepsia.

[18] Spoto G., Di Rosa G., Nicotera A.G. (2024). The Impact of Genetics on Cognition: Insights into Cognitive Disorders and Single Nucleotide Polymorphisms. J. Pers. Med..

[19] Feng X., Yang J., Chen N., Li S., Li T. (2025). Diagnostic yields of genetic testing and related benefits in infantile epileptic spasms syndrome: A systematic review and meta-analysis. Seizure.

[20] Dawson G., Rogers S., Munson J., Smith M., Winter J., Greenson J., Donaldson A., Varley J. (2010). Randomized, controlled trial of an intervention for toddlers with autism: The Early Start Denver Model. Pediatrics.

[21] Consoli C., Turriziani L., Antoci M., Lo Monaco M., Ceraolo G., Spoto G., Nicotera A.G., Di Rosa G. (2025). Sensory Phenotypes in Autism Spectrum Disorder Associated with Distinct Patterns of Social Communication, Repetitive and Restrictive Behaviors or Interests, and Comorbidities: A State-of-the-Art Review. Brain Sci..

[22] Riggs E.R., Andersen E.F., Cherry A.M., Kantarci S., Kearney H., Patel A., Raca G., Ritter D.I., South S.T., Thorland E.C. (2020). Technical standards for the interpretation and reporting of constitutional copy-number variants: A joint consensus recommendation of the American College of Medical Genetics and Genomics (ACMG) and the Clinical Genome Resource (ClinGen). Genet. Med. Off. J. Am. Coll. Med. Genet..

