# Supplementary material for: RFX3 Pathogenic Variants as a Rare Cause of Infantile Epileptic Spasms Syndrome
Source: Int J Mol Sci. 2026 Aug 13;27(16):7238. doi: 10.3390/ijms27167238 (PMC13513027; doi:10.3390/ijms27167238)
Supplement: Supplementary file 1 [file ijms-27-07238-s001.zip › ijms-4451210-supplementary.pdf]

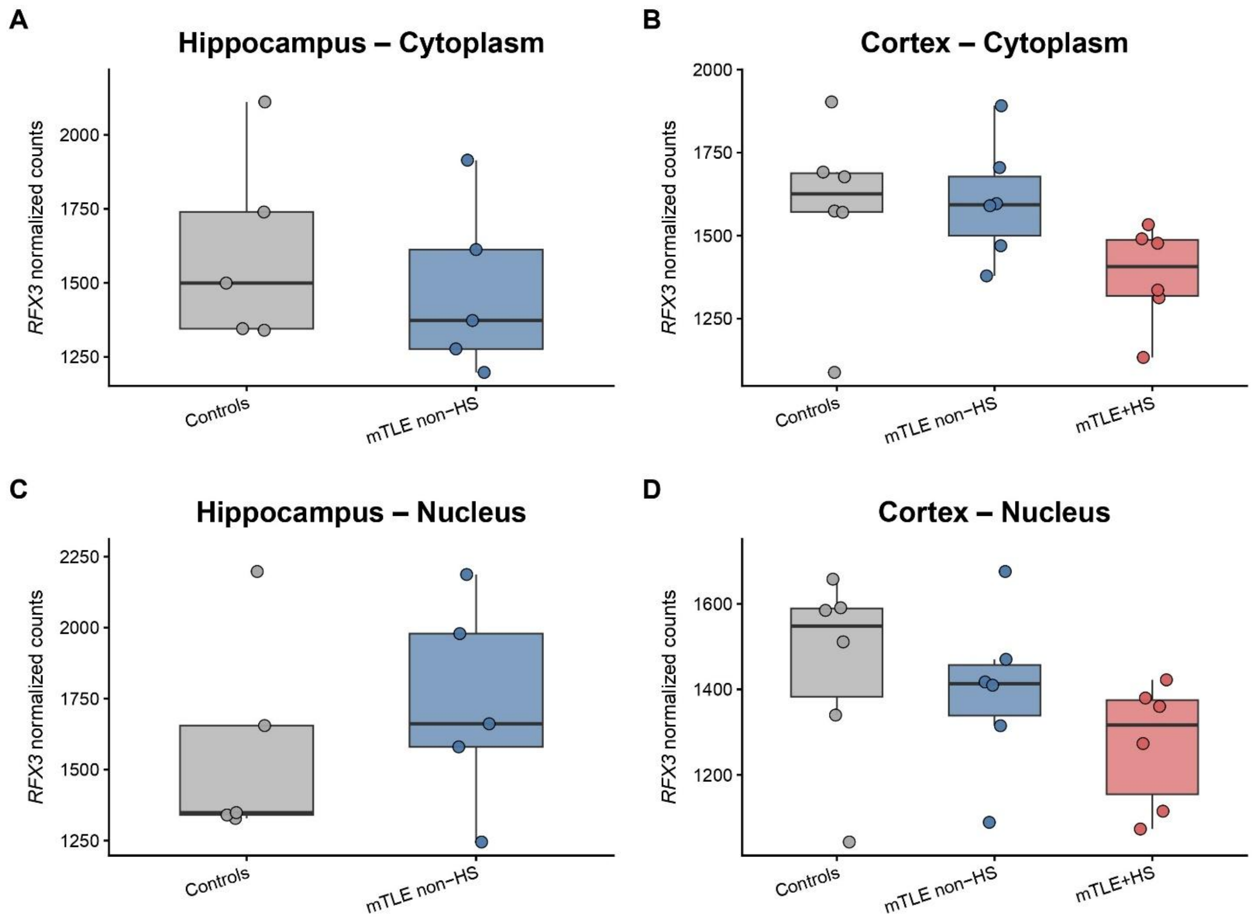

Figure S1. RFX3 expression in hippocampal and cortical tissue from patients with mesial temporal lobe epilepsy. RFX3 expression was evaluated in the publicly available GSE186334 RNA-sequencing dataset. Hippocampal samples included patients with mesial temporal lobe epilepsy without hippocampal sclerosis (mTLE non-HS; n = 5) and post-mortem controls (n = 5). Cortical samples included independent samples from patients with mTLE non-HS (n = 6), patients with mTLE and hippocampal sclerosis (mTLE+HS; n = 6), and post-mortem controls (n = 6). Cytoplasmic and nuclear RNA fractions were analyzed separately. Expression values are shown as DESeq2-normalized counts. Boxes represent the interquartile range, central lines indicate the median, whiskers extend to the most extreme values within 1.5 times the interquartile range, and dots represent individual samples. Differential expression analysis was performed on raw counts using DESeq2, and no statistically significant differences were detected in any comparison.

Table S1. Differential expression analysis of RFX3 in the publicly available GSE186334 RNA-sequencing dataset. Differential expression was assessed using DESeq2 on raw RNA-sequencing counts. Analyses were performed separately for each brain compartment, defined by tissue (hippocampus or cortex) and RNA fraction (cytoplasmic or nuclear). For hippocampal samples, only the comparison between mTLE without hippocampal sclerosis (mTLE non-HS) and post-mortem controls was performed because hippocampal samples from patients with hippocampal sclerosis were not available in the dataset. P-values were adjusted for multiple testing using the Benjamini–Hochberg procedure.

Compartment	Comparison	Cases (n)	Controls (n)	baseMean	log2FC	SE	Wald statistic	P-value	Adjusted P-value
Hip/Cyt	mTLE non-HS vs Controls	5	5	1540.98	-0.123	0.184	-0.670	0.503	0.927
Hip/Nuc	mTLE non-HS vs Controls	5	5	1652.16	0.136	0.206	0.662	0.508	0.919
Ctx/Cyt	mTLE non-HS vs Controls	6	6	1637.39	0.019	0.140	0.139	0.890	0.982
Ctx/Cyt	mTLE + HS vs Controls	6	6	1495.34	-0.200	0.141	-1.423	0.155	0.624
Ctx/Nuc	mTLE non-HS vs Controls	6	6	1375.16	-0.056	0.137	-0.408	0.683	0.928
Ctx/Nuc	mTLE + HS vs Controls	6	6	1495.43	-0.196	0.131	-1.491	0.136	0.526

Abbreviations: Hip, hippocampus; Ctx, cortex; Cyt, cytoplasmic RNA fraction; Nuc, nuclear RNA fraction; mTLE, mesial temporal lobe epilepsy; HS, hippocampal sclerosis; log2FC, log2 fold change; SE, standard error; FDR, false discovery rate.
